# Suprascapular nerve peripheral nerve stimulation for malignancy-related pain: A case series

**DOI:** 10.1016/j.inpm.2024.100421

**Published:** 2024-06-15

**Authors:** Cole Cheney, Jason Dauffenbach

**Affiliations:** Department of Pain Medicine, Mayo Clinic Health Systems, Mankato, MN, USA

**Keywords:** Suprascapular nerve, Peripheral nerve stimulation, Oncology pain, Neuromodulation

## Abstract

**Background:**

Blockade of the suprascapular nerve is an effective diagnostic tool in the workup and potential treatment of shoulder pain. For chronic shoulder pain, peripheral nerve stimulation has been shown to provide significant, sustained pain relief. However, no literature to date has described peripheral nerve stimulation for the treatment of oncologic shoulder pain.

**Objectives:**

We describe two cases of chronic oncologic-related shoulder pain that responded to posterior suprascapular peripheral nerve stimulator placement to facilitate future progress and discussion in the fields of peripheral nerve stimulation and oncology pain.

**Methods:**

Two subjects with chronic shoulder pain underwent ultrasound-guided peripheral nerve stimulation therapy at the suprascapular nerve.

**Results:**

At follow-up visits (30 and 98 days after procedure), both subjects reported greater than 50% pain relief as measured by the numerical rating scale (NRS).

**Conclusions:**

Peripheral nerve stimulator placement at the suprascapular nerve is a feasible procedure to treat oncologic shoulder pain via the described technique. Both subjects experienced clinically significant pain relief and decreased oral analgesic medication intake, and decreased medication-related side effects. This warrants further investigation including large comparative, prospective studies to better assess efficacy and safety of this approach.

## 1 Introduction

The shoulder is innervated by the lateral pectoral nerve, suprascapular nerve (SSN), axillary nerve, and lower subscapular nerve [1]. Diagnostic and therapeutic blocks of these nerves provide targeted and significant analgesia in the acute and chronic pain setting [[2], [3], [4]]. Recent shoulder radiofrequency ablation protocols recommend targeting the lateral pectoral, suprascapular, and axillary nerves. In addition, these nerves are a suitable target for peripheral nerve stimulator placement (PNS).

The SSN originates from the C5 and C6 nerve roots and arises from the upper trunk of the brachial plexus [5]. Tracing the course distally, it traverses the neck below the omohyoid muscle. It then courses upon the superior border of the scapula through the suprascapular canal, enters the suprascapular notch, and then enters the supraspinous fossa. Following this, the SSN travels along the lateral scapular border, through the spinoglenoid notch and caudally to the infraspinous fossa. The trapezius, supraspinatus, and infraspinatus cover the nerve posteriorly. The deltoid covers the nerve laterally. The nerve branches to both the supraspinatus and infraspinatus muscle (providing motor innervation). It provides afferent sensory innervation to the acromioclavicular and glenohumeral joint. The suprascapular artery and vein often course above or through the suprascapular notch adjacent to the nerve [6].

Several studies have described the successful use of PNS of the SSN for shoulder pain. A 2013 study targeted the SSN with PNS to successfully treat complex shoulder pain in a subject with history of superior labral tear from anterior to posterior (SLAP) repair, followed by 6 subsequent revision surgeries [7]. Subacromial impingement, adhesive capsulitis, biceps tendinopathy, acromioclavicular/glenohumeral joint arthritis, post-spinal cord injury shoulder pain, and acute post-operative pain are each conditions that have been managed by PNS at the shoulder [[8], [9], [10], [11]].

One case series demonstrated successful stimulation of the SSN for the treatment of C4-6 radiculopathy due to nerve root compression from metastatic renal cell carcinoma [12]. No literature to date has described the treatment of oncologic shoulder pain treated with a peripheral nerve stimulator at the SSN.

### 1.1 Ultrasound technique – posterior to anterior, medial to lateral approach

Due to the intensity of pain and imminent morbidity the decision was made not the pursue diagnostic block to prevent delays to care in one of the two subjects. A peripheral intravenous line is placed, and routine pre-operative antibiotics were administered within 60 minutes of procedure start. The subject is positioned in the upright seated position. The surgical area is sterilized with chlorhexidine solution twice and draped sterilely.

To localize the SSN using an ultrasound probe, we began by placing the probe at the suprascapular notch (out of plane relative the SSN). The superior transverse scapular ligament was visualized overlying the notch. Care was taken to identify the adjacent suprascapular artery and vein. The ideal needle entry point would be located posteriorly and medial to the suprascapular notch. The skin entry point was anesthetized with 1 ml of 1% lidocaine. A 19- gauge stimulating probe (SPRINT® PNS System MicroLead™, SPR Therapeutics, Inc, Cleveland, OH) was advanced within a 17-gauge percutaneous sleeve utilizing an in-plane technique until the probe was adjacent to the SSN. Of note, local anesthetic was not administered close to the nerve target (ensuring ability to capture response to test stimulation).

Multiple sites around the suprascapular notch were tested via stimulation. Subjects were asked to confirm paresthesia that covered their site of pain. Once confirmed, the sleeve was held in place while a 20-gauge MicroLead™ introducer (containing the lead) was inserted and advanced to the target depth. Stimulation parameters were again assessed and confirmed coverage of painful site. Stimulation was adjusted to evoke comfortable sensation in the region of pain. Stimulation was delivered at 100 Hz, and intensity could be adjusted from 1 to 100, corresponding to amplitudes of 0.2–30 mA and pulse durations of 10–200 us. A dual confirmation was obtained, first if the subject can perceive the stimulation, and second if they can confirm when the stimulation has been turned off.

The introducer-sleeve apparatus was withdrawn from the skin while manual pressure was applied at the lead entry site. A small amount of surgical glue was placed at the site of skin entry. The stimulator wire was then connected to a connector box and the entire site was covered in a bio-occlusive dressing.

In the recovery area, a more detailed programming occurred with device representative to determine an optimized amperage for that subject. Perception of stimulation can manifest as tingling, tapping, or buzzing. The ideal setting is one in which the subject feels a gentle sensation that is acceptable should it continue for a longer duration of time. The subjects returned to clinic 1-day post-procedure to confirm wound integrity and evaluate pain relief. Subjects were followed weekly via phone call with nursing until they achieved greater than 50% pain relief. Once this benchmark was achieved, they were next seen at 60-day system explant visit.

## 2 Methods

IRB approval was not required for this review. Next-of-kin informed consent was obtained for use of medical details and images.

## 3 Cases

### 3.1 Case 1: left shoulder pain due to multiple myeloma

A 67-year-old female with thoracic myelopathy, asthma, and multiple myeloma treated with three cycles of chemotherapy (Carfilzomib and Pomalidomide) presented to our pain clinic 12 months after developing severe left shoulder pain.

**Image 1:**
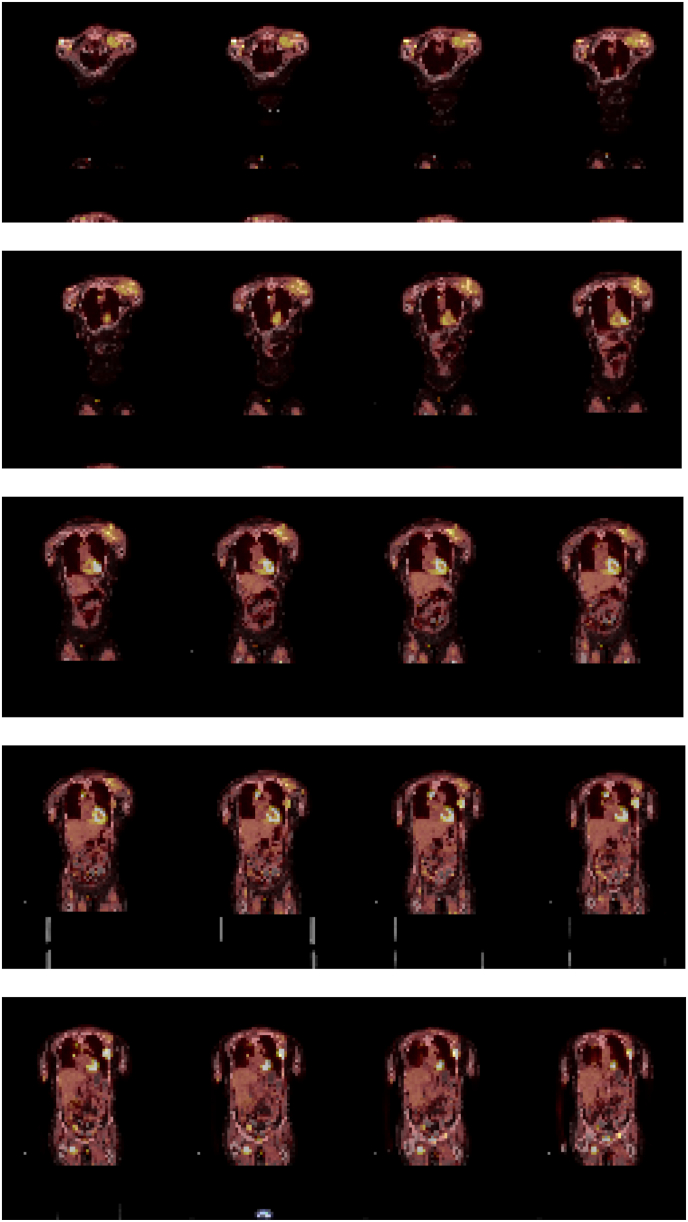
Patient 1 PET CT with increased tracer uptake at left shoulder.

**Image 2:**
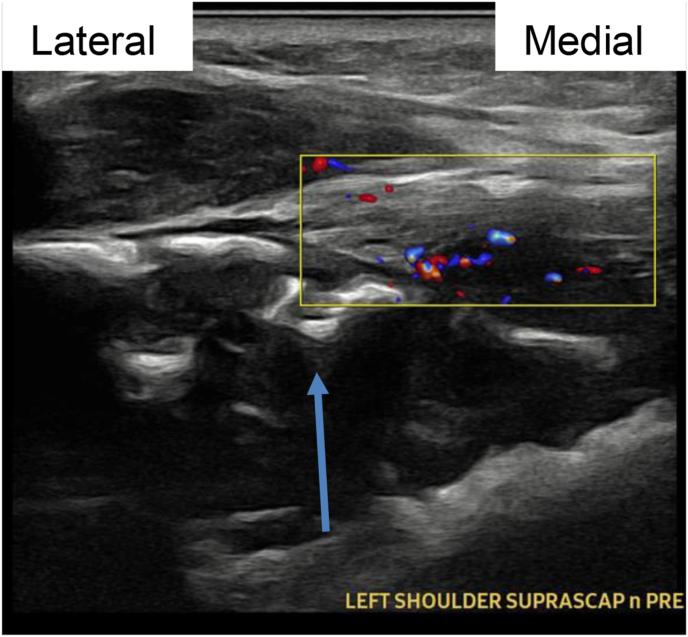
Patient 1 suprascapular nerve pre-procedure ultrasound image, short axis relative to scapular nerve. Doppler demonstrates hypervascularity near SSN. Blue arrow connotes suprascapular notch. (For interpretation of the references to color in this figure legend, the reader is referred to the Web version of this article.)

**Image 3:**
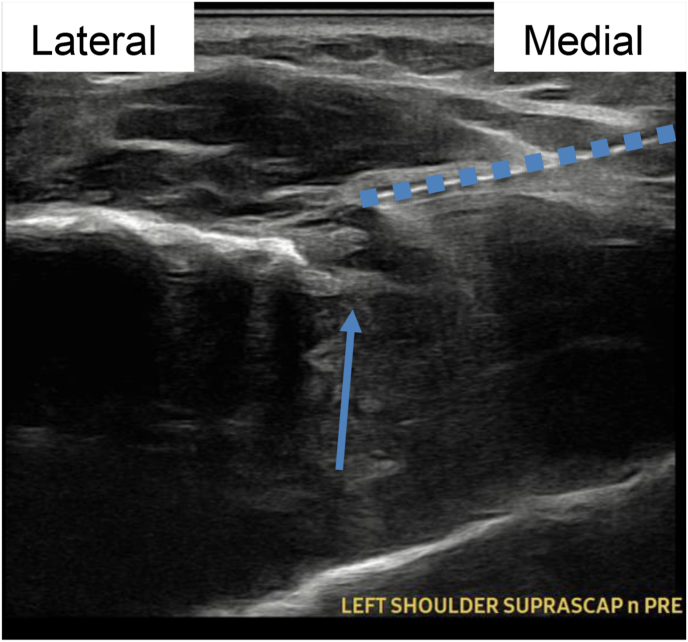
Patient 1 suprascapular nerve procedure ultrasound image during tract anesthetization with 25-gauge 1.5-inch hypodermic needle, short axis relative to scapular nerve. Blue arrow connotes suprascapular notch, blue dots connote needle. (For interpretation of the references to color in this figure legend, the reader is referred to the Web version of this article.)

**Image 4:**
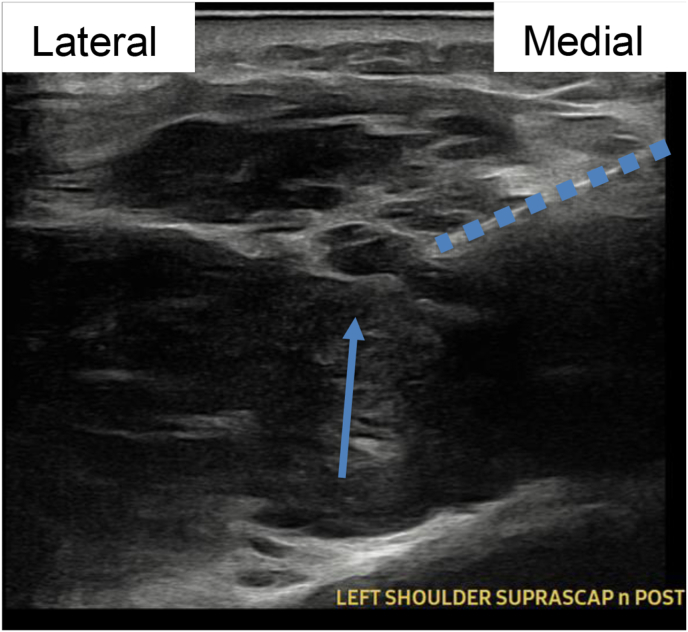
Patient 1 suprascapular nerve procedural ultrasound image, short axis relative to scapular nerve. Blue arrow connotes suprascapular notch. Dotted line connotes medial to lateral, superior to inferior PNS introducer trajectory. (For interpretation of the references to color in this figure legend, the reader is referred to the Web version of this article.)

At presentation she was on optimal doses of duloxetine and acetaminophen as well fentanyl 75 mcg/hour patch and oxycodone 15 mg every 4 hours as needed. Despite this, she described pain of ten out of ten on the Numerical Rating Scale (NRS). She described an ache and gnawing sensation in her posterior and lateral shoulder. Pain was exacerbated by forward flexion and overhead reach. X-ray shoulder demonstrated healing acromion base fracture. PET CT demonstrated hypermetabolic mass at left shoulder with maximum SUV of 10.95 in the left shoulder (growth from 8.47 the month prior) (Image 1). A peripheral nerve stimulation treatment was performed 3 months after initial presentation (Image 2, Image 3, Image 4). On follow-up four days after PNS placement, the subject reported pain level of 3/10 (NRS) and subjective report of 50% pain relief that was persistent through 30 days post-procedure. Shortly after, she was hospitalized for acute respiratory failure and discharged to hospice care. She expired one month and three days after system placement.

### 3.2 Case 2: Renal cell cancer with coracoid metastasis

**Image 5:**
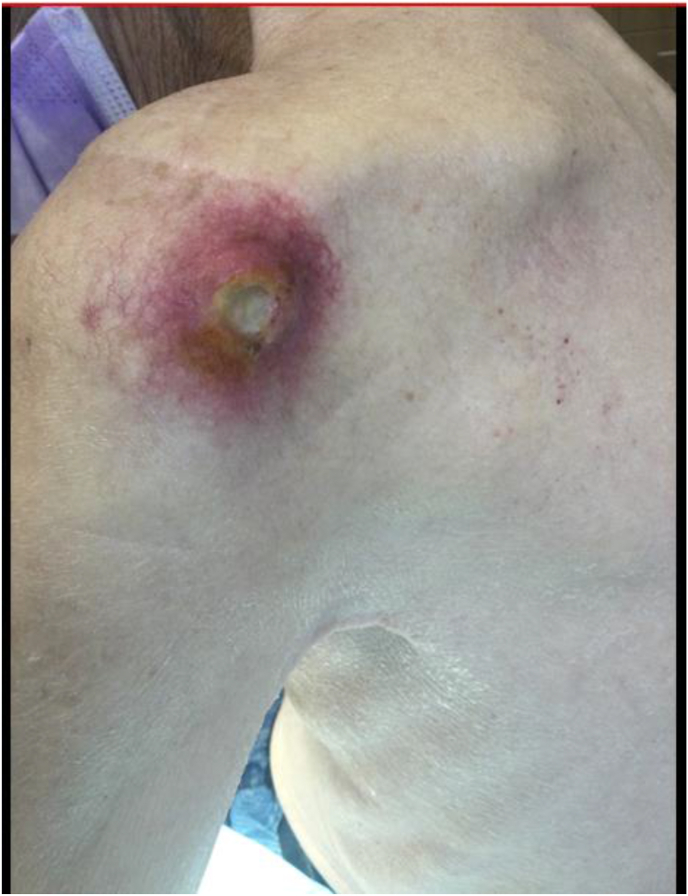
Patient 2 pre-procedural left shoulder with oncologic lesion.

A 67-year-old male with history of renal cell cancer with liver and bone metastasis. He underwent a number of oncologic treatments since 2005 after a right renal lesion was discovered. By 2015, he experienced sudden onset shoulder pain and locking that did not respond to physical therapy, oral medications, or glenohumeral joint injection. MRI demonstrated 2.7 x 2.4 × 1.8 cm mass centered in the coracoid process with extension into the anterosuperior glenoid. The mass grew to its largest dimensions of 5.8 cm × 5.3 cm x 4.7 cm in late 2016. A humeral head mass measuring 2.3 cm × 2.0 cm x 1.0 cm was discovered by 2021. By 2023, he had developed deformity, erythema, and ulceration of the overlying skin (Image 5). He tried gabapentin, nortriptyline, pregabalin, duloxetine, hydrocodone, and oxycodone. He found methadone 30 mg three times per day and hydromorphone 8 mg every 4 h to be the most effective pain treatment.

**Image 6:**
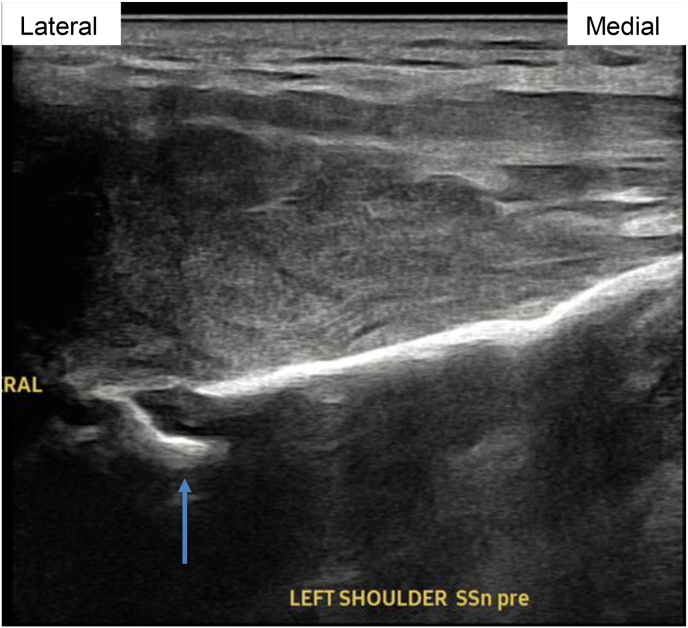
Patient 2 suprascapular nerve pre-procedure ultrasound image, short axis relative to scapular nerve. Blue arrow connotes suprascapular notch. (For interpretation of the references to color in this figure legend, the reader is referred to the Web version of this article.)

**Image 7:**
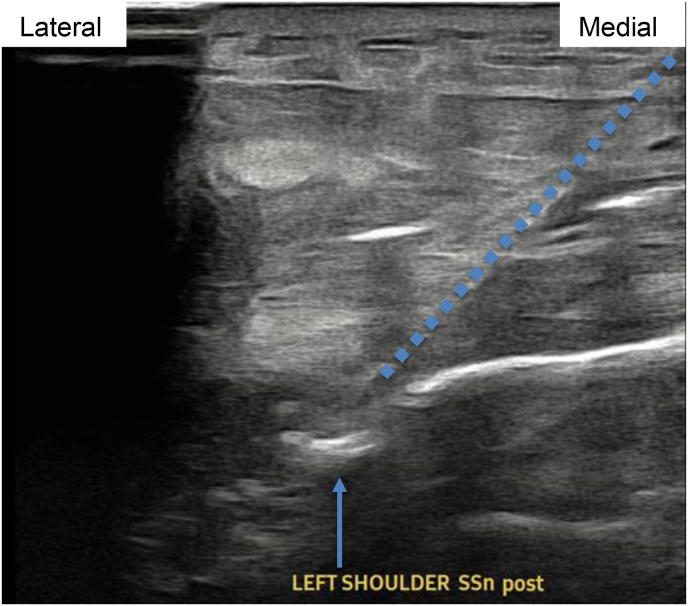
Patient 2 suprascapular nerve procedural ultrasound image, short axis relative to scapular nerve. Blue arrow connotes suprascapular notch. Dotted line connotes medial to lateral, superior to inferior PNS introducer trajectory. (For interpretation of the references to color in this figure legend, the reader is referred to the Web version of this article.)

**Image 8:**
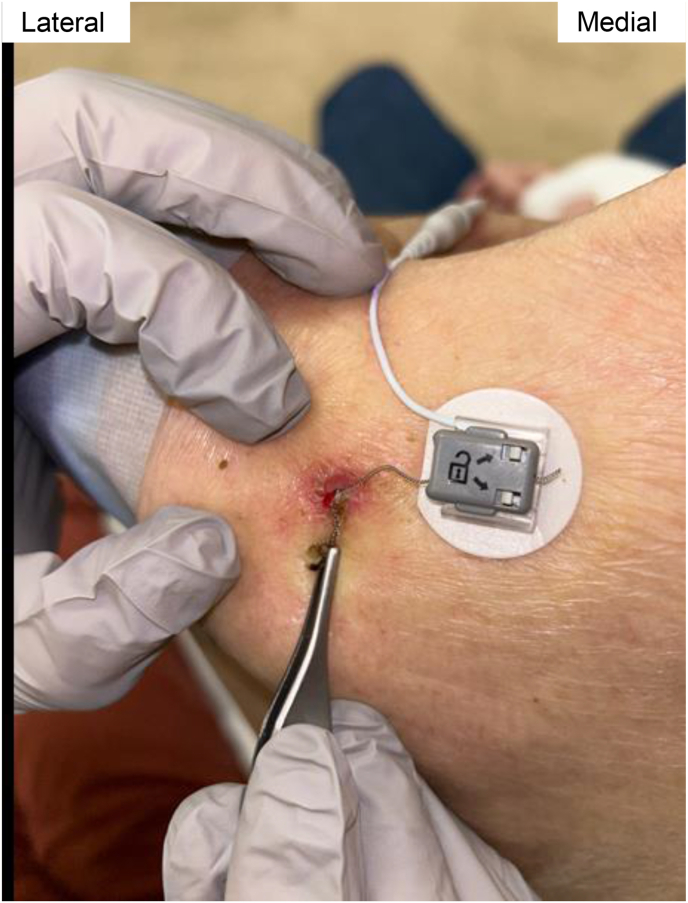
Patient 2 suprascapular nerve PNS system with associated infection.

A diagnostic left suprascapular nerve block with 2 ml of bupivacaine 0.5% and 2 ml of lidocaine 1.0% in early 2023 provided remarkable pain relief (NRS score of 5 improved to 1). An PNS system was placed shortly thereafter that provided 80% pain relief (Image 6, Image 7). Pre-procedure NRS peaked at 7/10 and post-procedure pain score was consistently reported at 0/10 throughout therapy. Previous analgesic therapy approximating 90 mg methadone and 48 mg oral hydromorphone decreased to a single 10 mg methadone tablet per day and approximately 16 mg of oral hydromorphone per day on average. Due to such profound effect, subject engaged in a risk and benefits discussion of removal vs. non-removal outside manufacturer recommendations (60-day maximum therapy). The subject opted to continue therapy outside recommended guidelines. The device remained in place for 98 days before concern for infection prompted removal (Image 8).

The subject's pain quickly returned status post device removal, prompting re-placement 2 months later. This time, he appreciated 100% pain relief at the shoulder and ceased of methadone at 1 month follow-up. Shortly after, he entered hospice care and expired 4 months post-procedure with PNS system in place.

## 4 Discussion

This series demonstrates a feasible and safe technique for peripheral nerve stimulator placement at the SSN for pain of oncologic etiology. All subjects received clinically significant pain relief without serious adverse events. In addition, they reported improvement in ADLs and decreased opioid analgesic use.

PNS involves the direct stimulation of nerves outside of the neuraxis and is an application of the gate control theory of pain [13]. Initially, PNS modalities involved surgical dissection to facilitate direct identification of the target nerve, followed by electrode placement. This procedure was limited to the treatment of traumatic or surgically induced neuropathies or complex regional pain syndrome type II. Use cases for PNS have significantly expanded due to needle-delivered lead placement systems reducing the invasiveness of electrode placement. Improved neuromodulatory waveforms are showing potential for increased response to therapy as well. Recent literature has highlighted the use of PNS for shoulder pain [8,9,11,14]. The cases discussed in this report uniquely demonstrates the use of an ultrasound guided needle based PNS delivery system and show the feasibility, efficacy, and simplicity of this procedure as an approach to address oncologic shoulder pain. At follow-up, all subjects reported satisfactory pain relief and functional improvements.

The suprascapular nerve was chosen as a target based on its coverage of the osseus joint structures in the shoulder that partially matched subjects' locations of pain and the authors' comfort with SSN interventions over that of the axillary or lateral pectoral nerve. The authors acknowledge that this approach did not cover the entirety of the subjects' shoulder pain. The peripheral nerve stimulation system used in this manuscript can power two leads; should either patient have expressed inadequate shoulder coverage, we intended to add a second lead based on subjects’ untreated pain distribution.

Several structures including the suprascapular artery and vein and the supraspinatus muscle occupy this space. In addition, profound neovascularization occurs near metastatic lesions that warrant particular care. Ultrasound guidance with color flow Doppler is a necessary tool in safe PNS placement.

We anticipate further development of PNS technology. Recent improvements have included reduced stimulator size and introduced high frequency settings all of which increase subject satisfaction and compliance with therapy. We sought to publish our approach to facilitate future progress in the field of peripheral nerve stimulation.

Further studies are warranted to evaluate the efficacy of this technique compared with other SSN placement techniques. In addition, future research is needed to evaluate the efficacy and safety profile of an needle based PNS system compared with conservative management, other minimally invasive techniques, and surgical interventions.

## 5 Conclusions

Peripheral nerve stimulator placement at the suprascapular nerve for oncologic shoulder pain is a feasible procedure via the described technique. All subjects experienced meaningful pain relief and reduced use of oral analgesic medications, though one subject experienced insertion site infection that required revision. This warrants further investigation including large comparative, prospective studies to better assess efficacy and safety of this approach.

## Funding

The authors have no sources of funding to declare for this manuscript.

## Contributorship

CC, JD contributed to the conception and design of the research, and critical revision of the manuscript. CC, JD contributed to the literature review and critique, and drafting of the manuscript. CC, JD contributed to the final approval of the manuscript.

## Declaration of competing interest

The authors declare the following financial interests/personal relationships which may be considered as potential competing interests:Cole Cheney consults for 2PD. A limb-loss pain device that is neither used, or applicable to this research. If there are other authors, they declare that they have no known competing financial interests or personal relationships that could have appeared to influence the work reported in this paper.
